# Disruption of retinal pigment epithelial cell properties under the exposure of cotinine

**DOI:** 10.1038/s41598-017-03283-x

**Published:** 2017-06-09

**Authors:** Xiao-Yu Zhang, Tsz Kin Ng, Mårten Erik Brelén, Kwok Ping Chan, Di Wu, Jasmine Sum Yee Yung, Di Cao, Yumeng Wang, Shaodan Zhang, Sun On Chan, Chi Pui Pang

**Affiliations:** 1 Department of Ophthalmology and Visual Sciences, The Chinese University of Hong Kong, Hong Kong, China; 2 Department of Ophthalmology, The Fourth People’s Hospital of Shenyang, Shenyang, China; 3 Shenyang Key Laboratory of Ophthalmology, Shenyang, China; 4 School of Biomedical Sciences, The Chinese University of Hong Kong, Hong Kong, China

## Abstract

Cigarette smoking is a major risk factor for age-related macular degeneration (AMD), in which progressive retinal pigment epithelial (RPE) cell degeneration is a major pathological change. Nicotine is a major biologically active component in cigarette smoke. It is continuously catabolized into cotinine, which has longer half-life and higher concentration in tissue cells and fluids. Here we hypothesized that continuous exposure of cotinine has more potent effects on human RPE cell properties than nicotine. Human RPE cell line (ARPE-19) was treated continuously with 1–2 µM of nicotine and/or cotinine for 7 days. RPE cells treated with 2 μM cotinine and nicotine-cotinine mixture has lower MTT signals without significant changes in cell apoptosis or integrity. Moreover, RPE cell migration was retarded under cotinine treatments, but not nicotine. Both nicotine and cotinine treatments attenuated the phagocytotic activity of RPE cells. In addition, cotinine and nicotine-cotinine mixture suppressed VEGF and IL-8 expression and upregulated TIMP-2 expression. Expressions of autophagy genes were upregulated by the cotinine treatment, whereas expressions of epithelial-to-mesenchymal transition markers were downregulated. In conclusion, our study, for the first time, demonstrated that cotinine, rather than nicotine, affects the properties of RPE cells *in vitro*, which could explain the smoking-induced RPE pathology.

## Introduction

Age-related macular degeneration (AMD) is a progressive retinal degenerative disease affecting more than 150 million people in the world^1^. Disruption of retinal pigment epithelium (RPE) and photoreceptors is one of the major pathological changes in AMD^2, 3^. Integrity of RPE is essential for regeneration of bleached visual pigment, generation and maintenance of inter-photoreceptor matrix and the Bruch membrane, selective transport of fluids and nutrients between photoreceptors and the choriocapillaries, and phagocytosis of photoreceptors^4, 5^.

The etiology of AMD is multi-factorial, among which genetic variants, such as *HTRA1* and *CFH* genes^6–8^, are critical determinants. Other factors include cigarette smoking, which is the most consistently replicated and modifiable environmental risk factor for AMD development. Our earlier studies have shown that cigarette smokers are 1.76-fold more prone to develop AMD than non-smokers^7, 8^. In addition, cigarette smoking also interacts with *HTRA1* rs11200638 polymorphism, superimposing the risk to 15.71 folds.

Cigarette smoke contains over 4,000 chemicals. Nicotine is a key component and the determinant for addiction to smoking^9, 10^. Moreover, it is also the major component in different cigarette replacements, including the recently popular electronic cigarette and nicotine patches. In human primary fetal RPE culture, exposure of 1 µM nicotine for 3 days changes RPE morphology without affecting cell proliferation, and reduces interleukin-8 (IL-8), metalloproteinase-2 (MMP2) and MMP9, but not vascular endothelial growth factor (VEGF) expression^11^. Similarly, exposure of 10 nM nicotine for 3 days did not induce ARPE-19 cell death or proliferation, but upregulated VEGF and downregulated PEDF expression^12^. In addition, one-day treatment of 10 or 0.1 mM nicotine did not affect ARPE-19 cell viability or caspase-3/7 activity^13^. Furthermore, nicotine (1, 10 or 100 µM) did not induce cell death in porcine RPE during the 7-day treatment, but decreased VEGF secretion and reduced the phagocytotic ability of the RPE^14^.

Under physiological conditions, nicotine, with a half-life of 2 hours, is continuously metabolized by hepatic cytochrome P450 enzyme CYP2A6 into cotinine^15^, which is the major metabolite of nicotine. The half-life of cotinine is 19 hours^16^. Plasma levels of nicotine and cotinine in daily cigarette smokers range from 0.08–0.15 μM and 1.02–1.73 μM, respectively^17, 18^. Cotinine has higher concentration and stays longer in the human body than nicotine. It possesses proven biological activities. Low concentration of cotinine (313 μg/ml or below) stimulated secretory epithelial cell viability, whereas high concentration of cotinine (1250 μg/ml or above) significantly decreased the total number of cells, metabolic activity as well as the secretory component^19^. Coherently, 10 nM cotinine is a mitogen for human vascular smooth muscle cells, but becomes toxic at higher concentrations^20^. However, the effect of cotinine on human RPE cells has yet to be examined. Here we hypothesized that cotinine has a more potent influence than nicotine on human RPE cells. We characterized the effects of continuous exposure of cotinine, with reference to nicotine, on the human RPE cells (ARPE-19), in terms of cell proliferation, cell apoptosis, cellular integrity, wound healing, angiogenic factor expression and phagocytotic activity. In addition, the mechanisms of the nicotine and cotinine effects were also investigated.

## Results

### The expression of nicotinic acetylcholine receptor subunits in ARPE-19 cells

**Figure 1:**
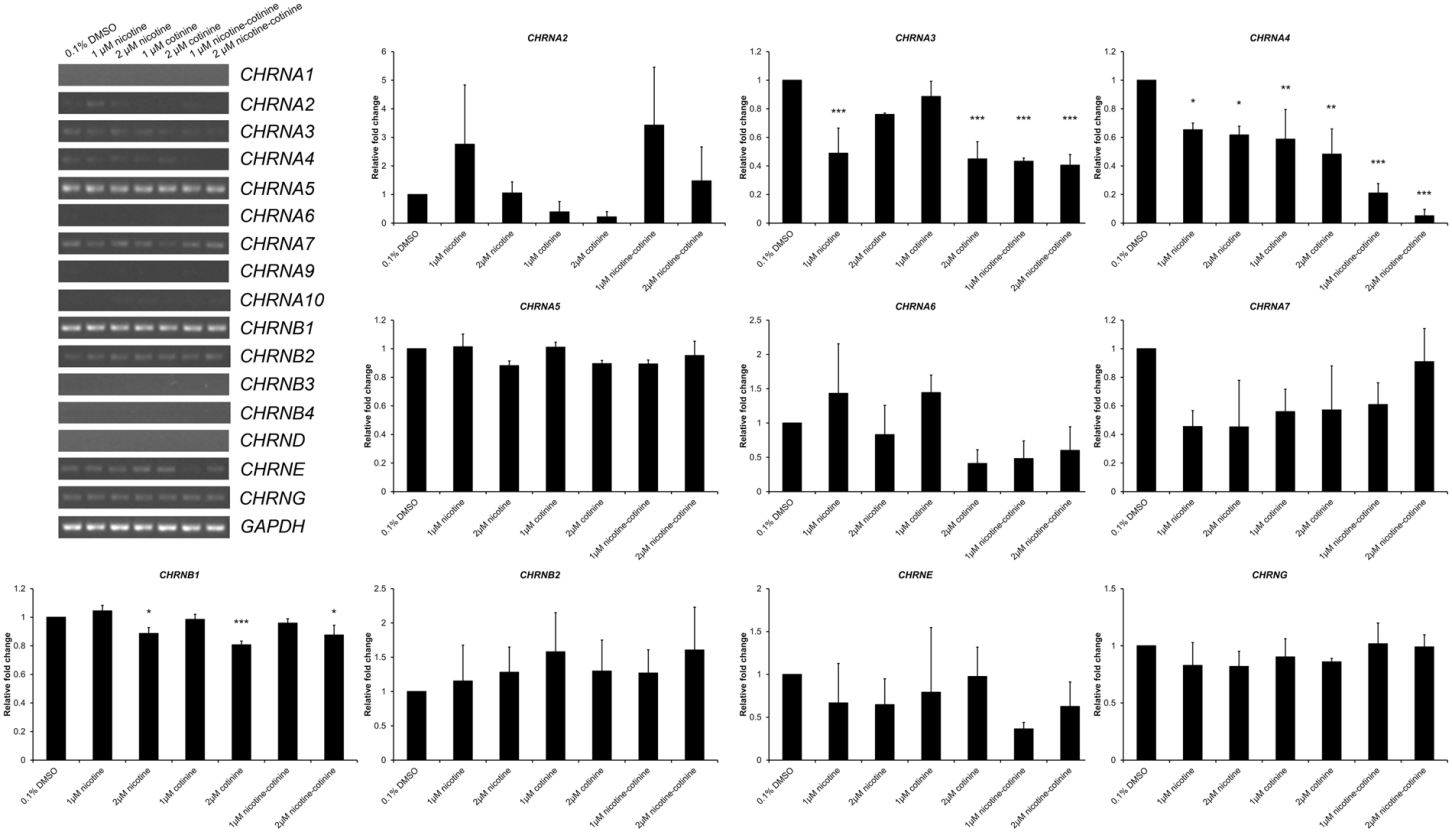
The expression of nicotinic acetylcholine receptor subunits in ARPE-19 cells. The gene expression of α (*CHRNA1-10*), β (*CHRNB1-4*) as well as δ (*CHRND*), ɛ (*CHRNE*) and γ (*CHRNG*) subunits nicotine acetylcholine receptor in ARPE-19 cells was determined by RT-PCR. ARPE-19 cells highly expressed *CHRNA5* and *CHRNB1*, moderately expressed *CHRNA7*, *CHRNB2*, *CHRNE* and *CHRNG*, and slightly expressed *CHRNA2*, *CHRNA3*, *CHRNA4* and *CHRNA6*. Low/no expressions of *CHRNA1*, *CHRNA9*, *CHRNA10*, *CHRNB3*, *CHRNB4* and *CHRND* were found. The expression of *CHRNA4* was dose-dependently and addictively downregulated across the nicotine and cotinine treatment groups, whereas the expression of *CHRNA3* was downregulated in the 1 µM nicotine, 2 µM cotinine and 1 and 2 µM nicotine-cotinine mixture groups. The expression of *CHRNB1* was downregulated in the 1 µM nicotine, 2 µM cotinine as well as 2 µM nicotine-cotinine mixture groups. ‘*’*p* < 0.05; ‘**’*p* < 0.01; ‘***’*p* < 0.001.

Nicotine has been shown to bind with the nicotinic acetylcholine receptor^21^. To understand the capability of ARPE-19 cells to respond to nicotine and cotinine, we examined the expression of nicotinic acetylcholine receptor gene by polymerase chain reaction (PCR). ARPE-19 cells highly expressed the α5 (*CHRNA5*) and β1 subunits (*CHRNB1*) of nicotinic acetylcholine receptor (Fig. 1), moderately expressed α7 (*CHRNA7*), β2 (*CHRNB2*), ɛ (*CHRNE*) and γ subunits (*CHRNG*), and slightly expressed α2 (*CHRNA2*), α3 (*CHRNA3*), α4 (*CHRNA4*) and α6 subunits (*CHRNA6*). Low/no expressions of α1 (*CHRNA1*), α9 (*CHRNA9*), α10 (*CHRNA10*), β3 (*CHRNB3*), β4 (*CHRNB4*) and δ subunits (*CHRND*) were found in ARPE-19 cells. The expression of α4 subunit was dose-dependently and addictively downregulated across the nicotine and cotinine treatment groups, whereas the α3 subunit expression was downregulated in the 1 µM nicotine, 2 µM cotinine and 1 and 2 µM nicotine-cotinine mixture groups. Furthermore, the expression of β1 subunit was downregulated in the 1 µM nicotine, 2 µM cotinine as well as 2 µM nicotine-cotinine mixture groups. These results indicated that ARPE-19 cells could be responded to nicotine and/or cotinine.

### The effect of nicotine and cotinine on RPE cell survival

**Figure 2:**
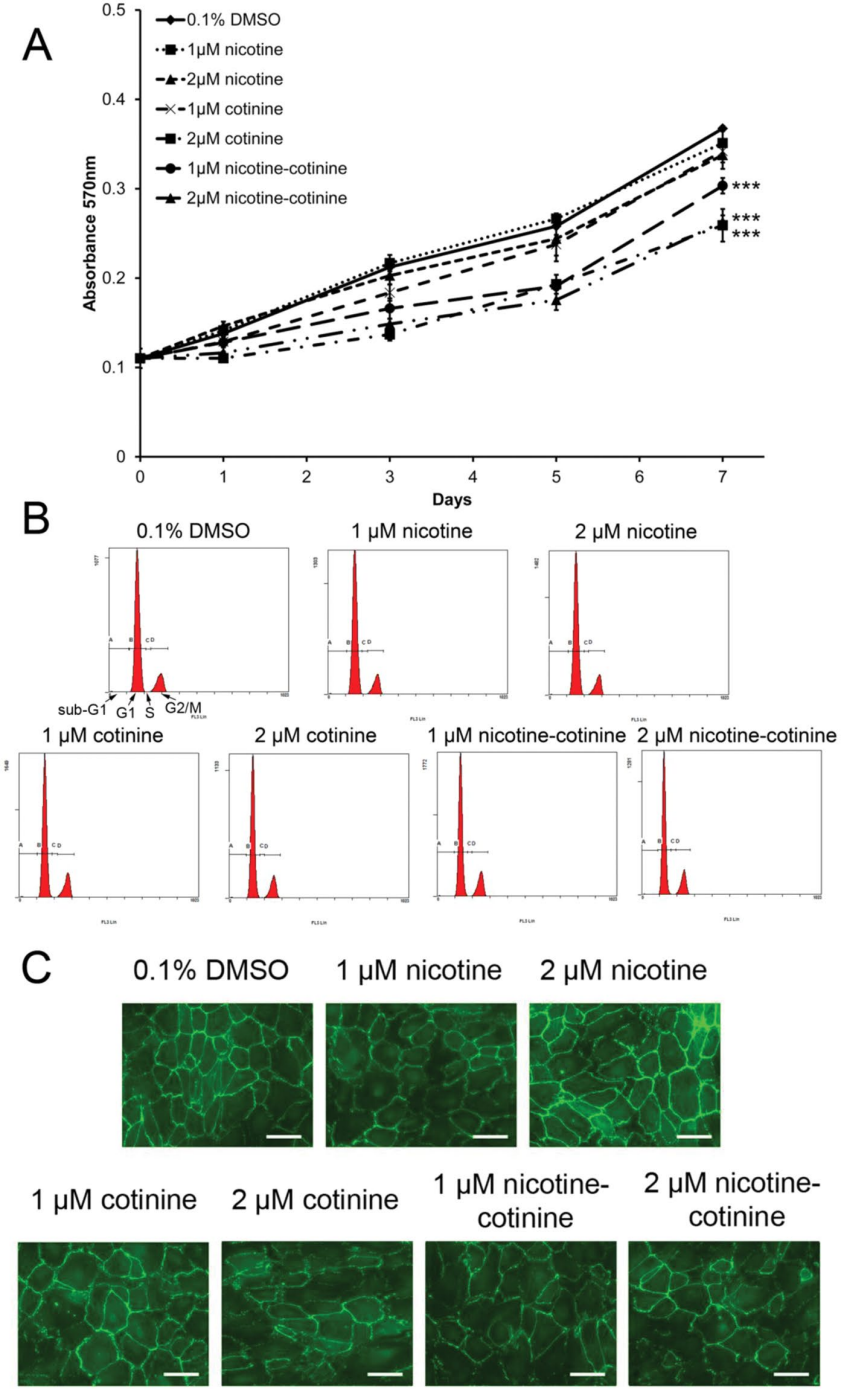
The effect of nicotine and cotinine on RPE cell survival. (**A**) Cell viability of ARPE-19 cells with nicotine and/or cotinine treatments was assessed by MTT assay. Significant reduction in ARPE-19 cell viability was observed in 2 µM cotinine as well as 1 and 2 µM nicotine-cotinine mixture compared to the vehicle control group (0.1% DMSO). (**B**) Cell cycle analysis was evaluated by propidium iodide staining and flow cytometry analysis. There was no significant difference in sub-G1, G1, S or G2/M populations among the nicotine, cotinine or nicotine-cotinine mixture groups compared to the vehicle control group (0.1% DMSO). A: sub-G1 phase; B: G1 phase; C: S phase; D: G2/M phase. **(C)** RPE cell integrity was determined by immunofluorescence analysis of tight junction protein (ZO-1; green). The cell number, cell morphology and membrane organization of ZO-1 were similar in the nicotine and cotinine treatment groups with the control group. Scale bars: 50 µm.

RPE cell proliferation under nicotine and cotinine treatments was analyzed by MTT (3-(4,5-dimethylthiazol-2-yl)-2,5-diphenyltetrazolium bromide) assay that 2 µM cotinine and 1 and 2 µM nicotine-cotinine mixture significantly reduced MTT signals in ARPE-19 cells by 25.19%, 25.97% and 32.04% at Day 5 and by 29.49%, 17.42% and 28.40% at Day 7, respectively, compared to the vehicle control (0.1% DMSO; *p* < 0.001; Fig. 2A). No significant difference was detected in 1 and 2 µM nicotine and 1 µM cotinine treatment groups.

To delineate the reduction in MTT signals, we performed flow cytometry analyses to evaluate cell apoptosis after nicotine and cotinine treatments. There was no significant difference in sub-G1 population among the nicotine, cotinine or nicotine-cotinine mixtures (0.80 ± 0.29% – 1.05 ± 0.55%; *p* > 0.05) compared to the vehicle control (1.14 ± 0.66%; Fig. 2B and Table 1), indicating that neither nicotine nor cotinine would cause RPE cell apoptosis. Apart from the sub-G1 population, there was also no significant difference in G1, S and G2/M populations among the nicotine, cotinine or nicotine-cotinine mixtures (G1: 81.32 ± 4.16% – 85.92 ± 2.90%; S: 1.06 ± 0.44% – 2.16 ± 1.39%; G2/M: 11.92 ± 3.29% – 16.36 ± 3.77%; *p* > 0.05) compared to the vehicle control (G1: 85.22 ± 3.32%; S: 1.30 ± 0.62%; G2/M: 12.24 ± 3.56%; Table 1).

**Table 1:** Cell cycle analysis of ARPE-19 cells treated with nicotine and/or cotinine.

	sub-G1 phase (*p* value)	G1 phase (*p* value)	S phase (*p* value)	G2/M phase (*p* value)
0.1% DMSO	1.14 ± 0.66% (−)	85.22 ± 3.32% (−)	1.30 ± 0.62% (−)	12.24 ± 3.56% (−)
1 µM nicotine	0.95 ± 0.38% (*p*_*post-hoc*_ = 0.998)	85.52 ± 2.04% (*p*_*post-hoc*_ = 1.000)	1.57 ± 1.01% (*p*_*post-hoc*_ = 1.000)	11.94 ± 2.92% (*p*_*post-hoc*_ = 1.000)
2 µM nicotine	0.94 ± 0.49% (*p*_*post-hoc*_ = 0.997)	85.92 ± 2.90% (*p*_*post-hoc*_ = 1.000)	1.09 ± 0.57% (*p*_*post-hoc*_ = 1.000)	11.92 ± 3.29% (*p*_*post-hoc*_ = 1.000)
1 µM cotinine	1.05 ± 0.55% (*p*_*post-hoc*_ = 1.000)	81.32 ± 4.16% (*p*_*post-hoc*_ = 0.687)	1.12 ± 0.40% (*p*_*post-hoc*_ = 1.000)	16.36 ± 3.77% (*p*_*post-hoc*_ = 0.744)
2 µM cotinine	1.01 ± 0.50% (*p*_*post-hoc*_ = 1.000)	82.79 ± 3.64% (*p*_*post-hoc*_ = 0.950)	1.06 ± 0.44% (*p*_*post-hoc*_ = 1.000)	14.88 ± 3.51% (*p*_*post-hoc*_ = 0.958)
1 µM nicotine-cotinine	0.88 ± 0.44% (*p*_*post-hoc*_ = 0.987)	81.74 ± 4.01% (*p*_*post-hoc*_ = 0.783)	2.16 ± 1.39% (*p*_*post-hoc*_ = 0.835)	15.02 ± 4.97% (*p*_*post-hoc*_ = 0.947)
2 µM nicotine-cotinine	0.80 ± 0.29% (*p*_*post-hoc*_ = 0.955)	81.96 ± 3.66% (*p*_*post-hoc*_ = 0.829)	2.06 ± 1.39% (*p*_*post-hoc*_ = 0.897)	15.01 ± 4.78% (*p*_*post-hoc*_ = 0.947)

To verify the cell apoptosis results, we examined the integrity of RPE cells, which reflects the maintenance of the RPE cell sheet, after 7-day nicotine and cotinine treatments. Immunofluorescence analysis of tight junction protein (zonula occludens protein 1, ZO-1) showed that the number and morphology of the ARPE-19 cells as well as the cell membrane organization of ZO-1 protein were maintained even after the nicotine and cotinine treatments (Fig. 2C). The results suggested that nicotine and cotinine do not cause RPE cell loss or affect its cell integrity.

### The effect of nicotine and cotinine on RPE cell migration

**Figure 3:**
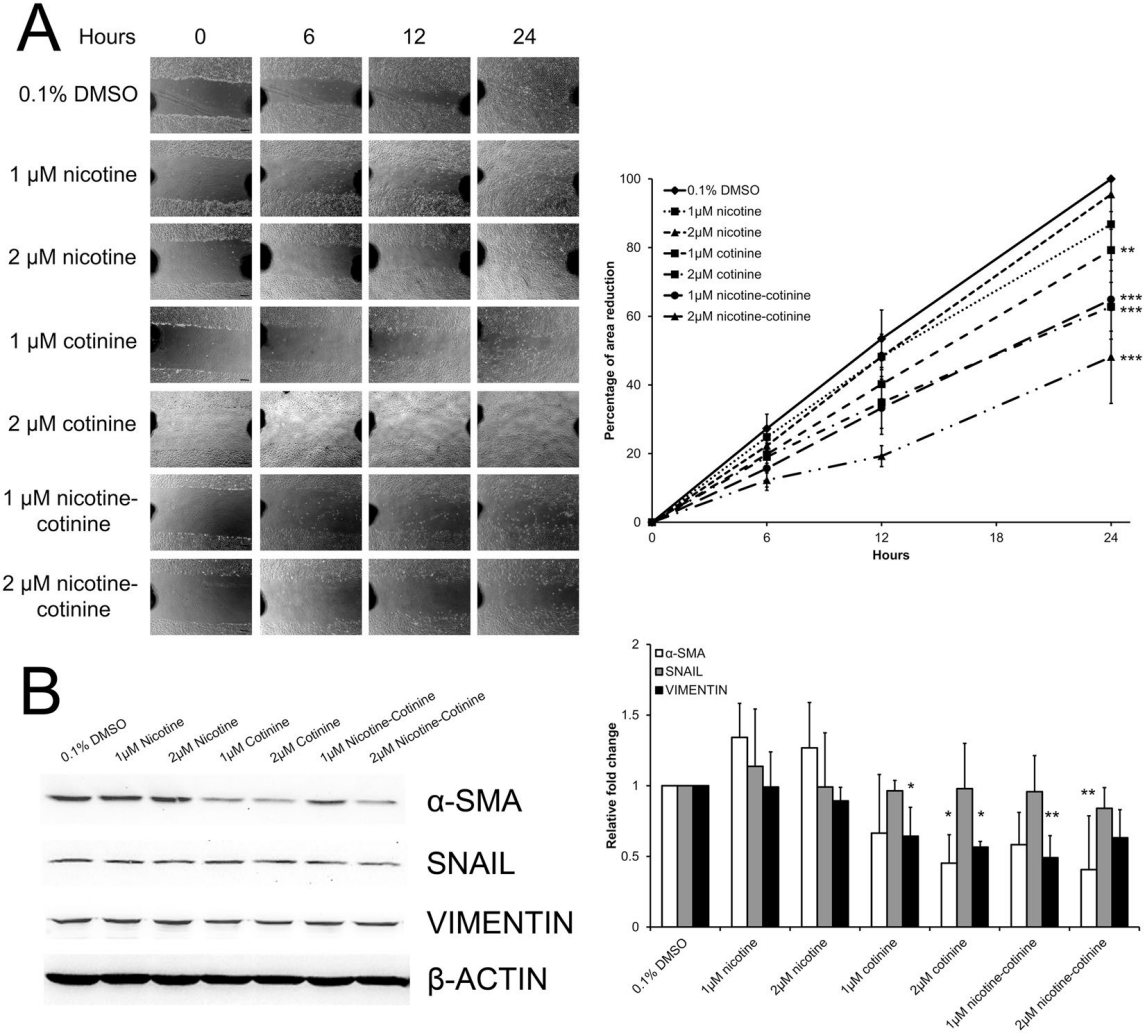
The effect of nicotine and cotinine on RPE cell migration. (**A**) ARPE-19 cell migration after different nicotine and/or cotinine treatments was evaluated by scratch wound assay. Images were taken at time 0, 6, 12 and 24 hours. The wound area was measured by Image J software. The percentage migration was calculated by the average area reduction at 6, 12 or 24-hour as compared to time 0. The data represented was the mean of triplicated experiments ± standard deviation. Scale bar: 100 μm. ‘**’*p* < 0.01; ‘***’*p* < 0.001. (**B**) The expression of epithelial-to-mesenchymal transition (EMT) marker protein (α-SMA, SNAIL and VIMENTIN) in ARPE-19 cell after different nicotine and/or cotinine treatments was evaluated by immunoblotting. Compared to the vehicle control group, the expression of α-SMA was significantly reduced in the 2 μM cotinine and 2 μM nicotine-cotinine mixture groups, whereas the expression of VIMENTIN was significantly decreased in the 1 μM and 2 μM cotinine as well as 1 μM nicotine-cotinine mixture groups. β-actin was used as housekeeping protein for normalization. ‘*’*p* < 0.05; ‘**’*p* < 0.01.

RPE cell migration is important in wound healing. We evaluated the migration of ARPE-19 cells by the scratch wound assay. Scratch wound was induced after the 7-day nicotine and/or cotinine treatment. The migratory ability of ARPE-19 cells was not affected by the 1 μM and 2 μM nicotine treatment (Fig. 3A). ARPE-19 cell migration was significantly retarded dose-dependently in the 1 μM and 2 μM cotinine as well as 1 μM and 2 μM nicotine-cotinine mixtures by 20.74%, 37.23%, 35.14% and 51.85%, respectively, at 24 hours, when compared to the vehicle control group (*p* < 0.001). These results showed that RPE cell migration is inhibited by cotinine but not nicotine.

In order to delineate the mechanism of the retarded cell migration, we determined the protein expression of migration-related epithelial-to-mesenchymal transition (EMT) markers, including α-smooth muscle action (α-SMA), SNAIL and VIMENTIN. Immunoblotting analysis showed that the expression of α-SMA was significantly reduced in the 2 μM cotinine and 2 μM nicotine-cotinine mixtures by 2.21 and 2.46 folds, respectively (*p* < 0.05), compared to the vehicle control (Fig. 3B). Similarly, the expression of VIMENTIN was also significantly decreased in the 1 μM and 2 μM cotinine, as well as in 1 μM nicotine-cotinine mixtures by 1.56, 1.77 and 2.04 folds, respectively (*p* < 0.05). However, no differential expression was found for SNAIL. The results suggested that the retarded RPE cell migration by cotinine was associated with the reduced expression of α-SMA and VIMENTIN.

### The effect of nicotine and cotinine on angiogenic factor expression in RPE cells

**Figure 4:**
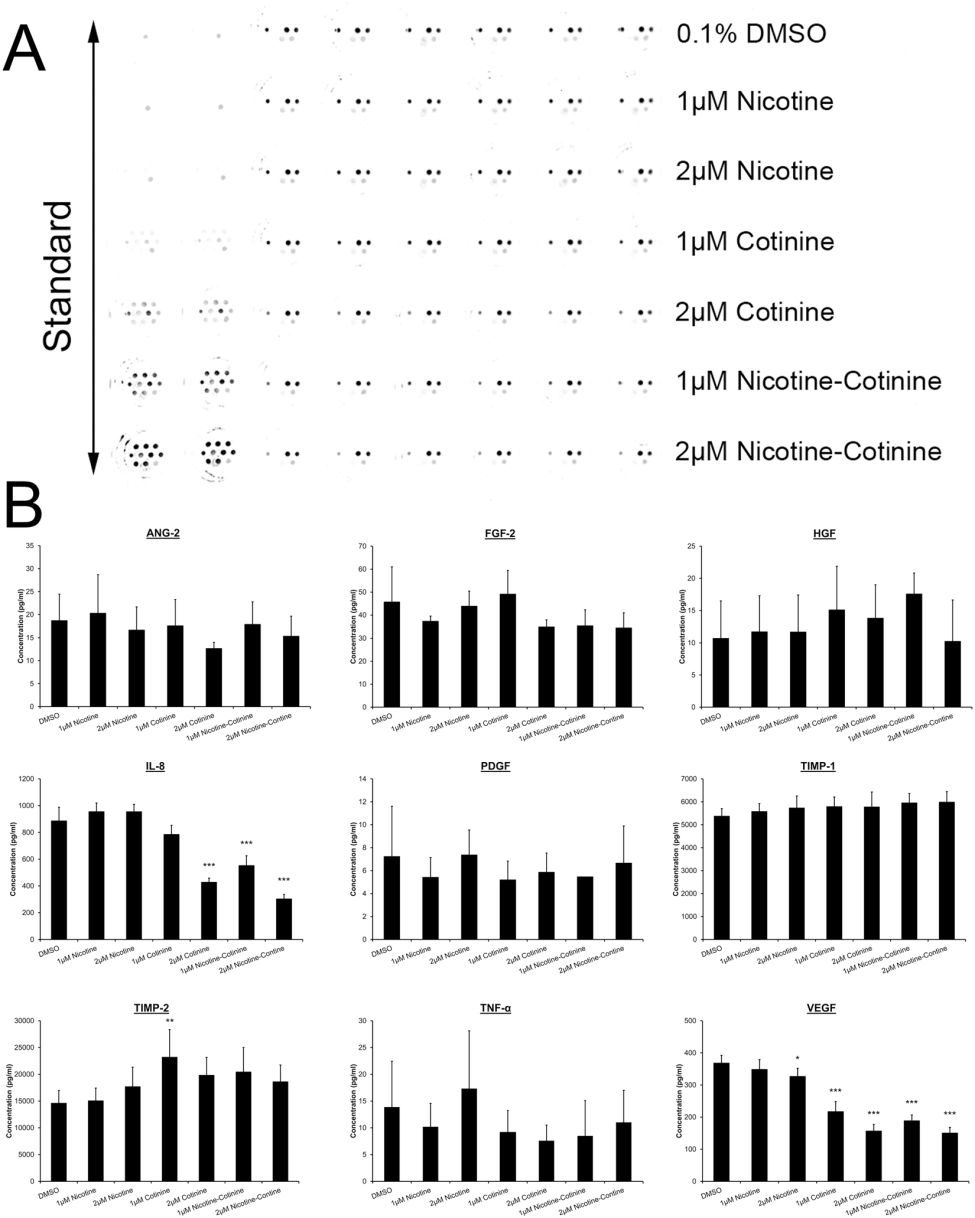
The effect of nicotine and cotinine on angiogenic factor expression in RPE cells. The expression of 9 human angiogenic factors was determined by a multiplex ELISA array. (**A**) Top (from left to right): ANG-2, FGF and HGF; Middle (from left to right): PDGF, IL-8, TIMP-1 and TIMP-2; Bottom (from left to right): TNFα, VEGF and reference spot. (**B**) Quantification of the expression of the 9 human angiogenic factors according to the standard curves for each factor. The relative expression levels were compared to that of the vehicle control group. ‘*’*p* < 0.05; ‘**’*p* < 0.01; ‘***’*p* < 0.001.

The angiogenic factors secreted by RPE cells are critical in the development of choroidal neovascularization (CNV). We collected the supernatant of cultured ARPE-19 cells after 7-day nicotine and/or cotinine treatment. The protein expression of total 9 angiogenic factors, including angiopoietin-2 (ANG-2), basic fibroblast growth factor (FGF), hepatocyte growth factor (HGF), interleukin-8 (IL-8), platelet-derived growth factor (PDGF), TIMP metallopeptidase inhibitor 1 (TIMP-1), TIMP-2, tumor necrosis factor α (TNF-α) and VEGF, were examined by the multiplex assay (Fig. 4A). Significant reduction in IL-8 expression was detected in the 2 μM cotinine as well as 1 μM and 2 μM nicotine-cotinine mixtures (426.98 ± 31.44, 551.31 ± 74.22 and 302.71 ± 33.20 pg/ml, respectively) when compared to the vehicle control (885.53 ± 103.25 pg/ml, *p* < 0.001; Fig. 4B). Similarly, VEGF expression was also significantly decreased in the 2 μM nicotine, 1 μM and 2 μM cotinine as well as 1 μM and 2 μM nicotine-cotinine mixtures (326.24 ± 25.77, 216.83 ± 31.58, 156.45 ± 20.87, 188.39 ± 18.28, 150.18 ± 18.06 pg/ml, respectively) in a dose-dependent manner, when compared to the vehicle control (367.96 ± 24.47 pg/ml; *p* < 0.05). In contrast, increased TIMP-2 expression was found in the 1 μM cotinine treatment group (23179.80 ± 5179.48 pg/ml vs control: 14577.69 ± 2380.89 pg/ml; *p* < 0.01). These results indicated that cotinine reduces pro-angiogenic factor expression and enhance anti-angiogenic factor expression.

### The effect of nicotine and cotinine on RPE phagocytotic activity

**Figure 5:**
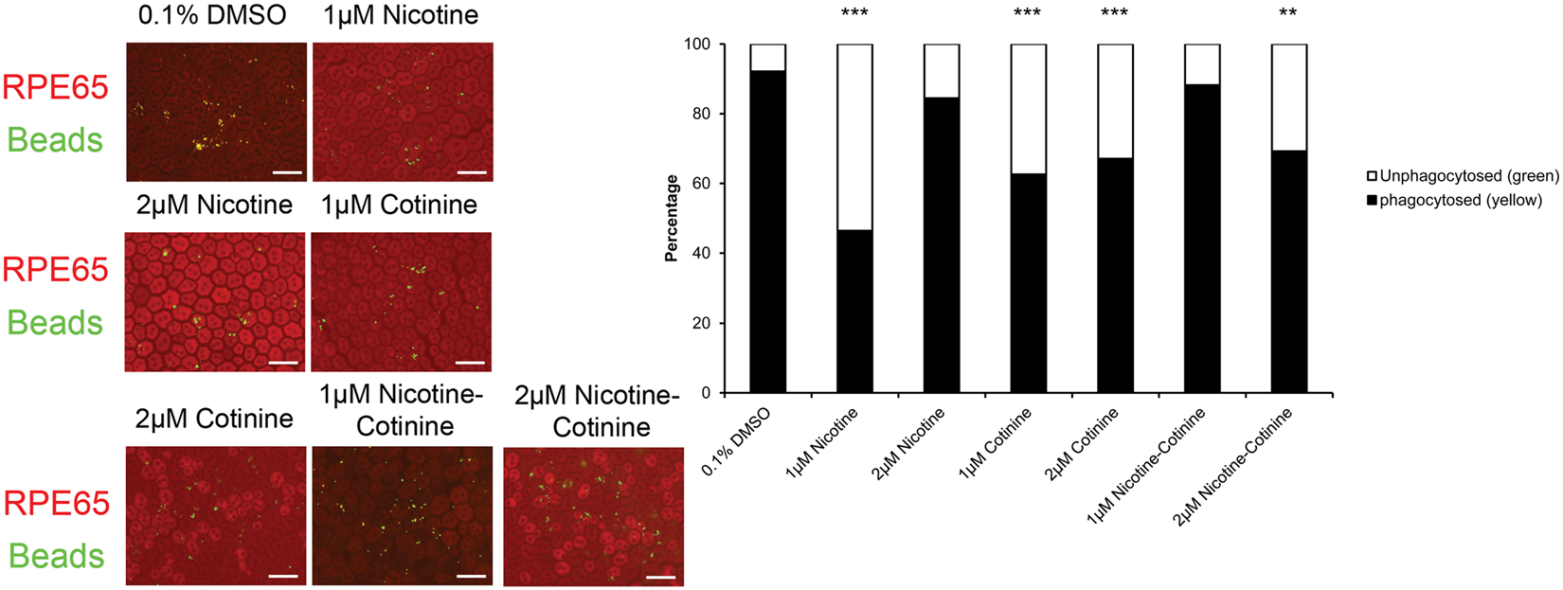
The effect of nicotine and cotinine on RPE phagocytotic activity. Photoreceptor outer segment phagocytosis was performed on the rat RPE explant culture. RPE cells were visualized by the immunofluorescence signal of RPE65 (Red), whereas FITC-labeled latex beads with rat POS were opsonizied. After 5-day nicotine and cotinine treatment, the phagocytotic activity of RPE cells was measured. The beads phagocytosed by RPE were in yellow, where those not phagocytosed by RPE were in green. The numbers of yellow and green beads in different treatment groups were counted and compared to that of the vehicle control. The RPE phagocytotic activity was significantly reduced in 1 μM nicotine, 1 and 2 μM cotinine and 2 μM nicotine-cotinine treatment groups. Scale bars: 50 µm. ‘**’*p* < 0.01; ‘***’*p* < 0.001.

To evaluate the phagocytotic function of RPE cells, we performed the photoreceptor outer segment (POS) phagocytosis analysis on the explant culture of RPE isolated from rats, which preserves the morphology and polarity of native RPE cells (Fig. 5). We have previously demonstrated that phagocytosed beads would show yellow fluorescence signal and those not phagocytosed would be in green signal^22^. After 5-day nicotine and cotinine treatment, the phagocytotic activity of rat RPE cells on rat POS was significantly reduced in 1 μM nicotine (46.43% phagocytosed beads; *p* < 0.001), 1 μM cotinine (62.59% phagocytosed beads; *p* < 0.001), 2 μM cotinine (67.09% phagocytosed beads; *p* < 0.001) and 2 μM nicotine-cotinine treatments (69.23% phagocytosed beads; *p* < 0.01), compared to the vehicle control (92.18% phagocytosed beads). Our results demonstrated that continuous nicotine and/or cotinine treatment would attenuate the phagocytotic activity of RPE cells.

### The effect of nicotine and cotinine on stress-related marker expression

**Figure 6:**
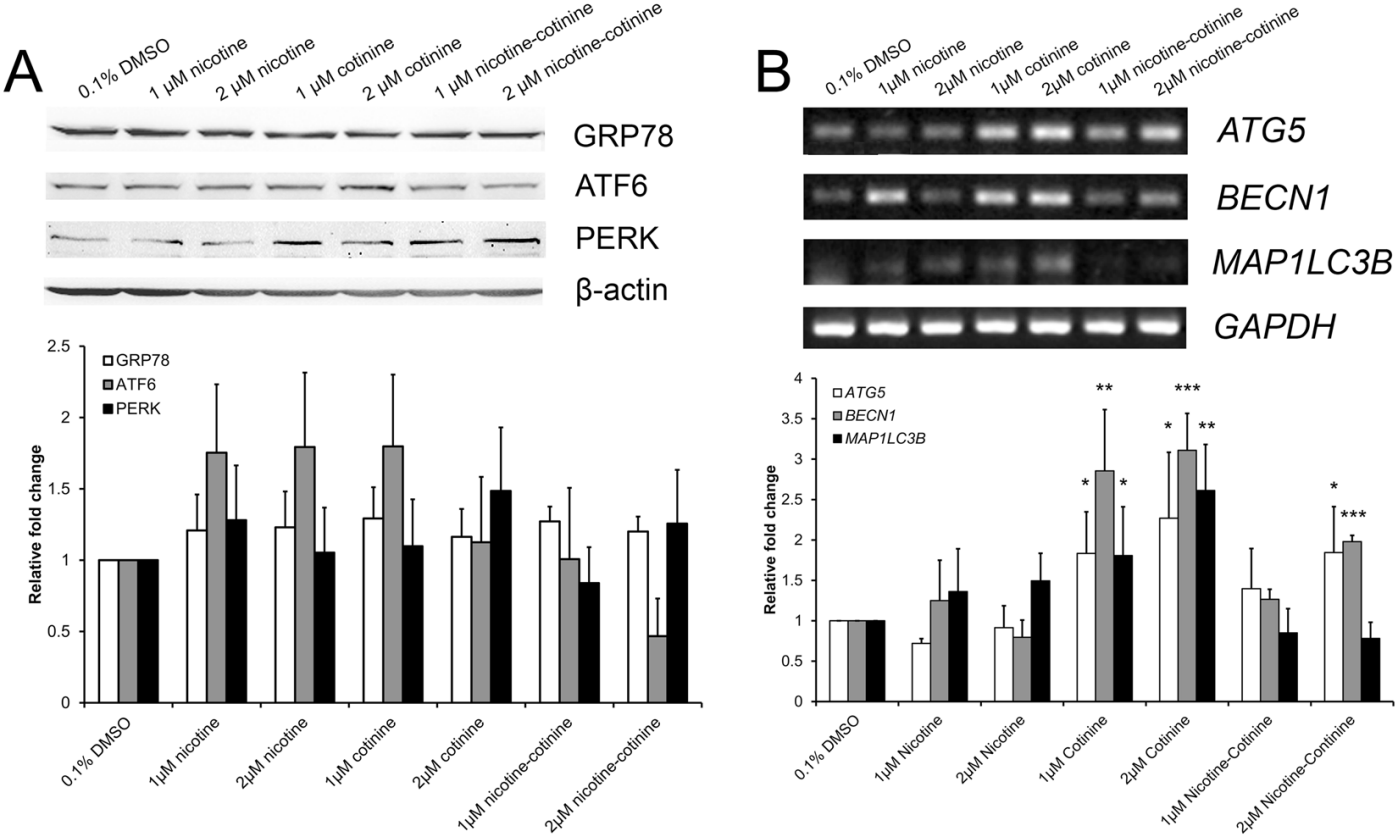
The effect of nicotine and cotinine on ER stress and autophagy pathway marker expression. (**A**) The expression of endoplasmic reticulum (ER) stress response protein (GRP78, ATF6 and PERK) in ARPE-19 cell after different nicotine and/or cotinine treatments was evaluated by immunoblotting. Neither nicotine nor cotinine caused any significant differential expression in these ER stress markers. β-actin was used as housekeeping protein for normalization. (**B**) The expression of autophagy pathway gene (*ATG5*, *BECN1* and *MAP1LC3B*) in ARPE-19 cells after different nicotine and/or cotinine treatments was analyzed by semi-quantitative PCR. 1 μM and 2 μM cotinine treatment groups significantly increased the expression of the 3 autophagy pathway genes, whereas 2 μM nicotine-cotinine treatment group only increased the expression of *ATG5* and *BECN1* genes. Housekeeping gene (*GAPDH*) was used for normalization. The relative expression levels were compared to that of the vehicle control group. ‘*’*p* < 0.05; ‘**’*p* < 0.01; ‘***’*p* < 0.001.

Since smoke exposure induces endoplasmic reticulum (ER) stress in mouse RPE cells^23^, we determined the protein expression of ER stress markers activating transcription factor 6 (ATF6), 78 kDa glucose-regulated protein (GRP78) and pancreatic ER kinase (PERK), in the nicotine and/or cotinine-treated ARPE-19 cells. Immunoblotting analysis showed that nicotine and/or cotinine did not cause any significant differential expression in these ER stress markers (Fig. 6A), indicating that neither nicotine nor cotinine induces ER stress response in RPE cells.

Apart from the ER stress response, we also examined the gene expression in the autophagy pathway, such as autophagy protein 5 (*ATG5*), Beclin-1 (*BECN1*) and microtubule-associated proteins 1 A/1B light chain 3B (*MAP1LC3B*) genes, after 7-day of nicotine and/or cotinine treatment. Semi-quantitative polymerase chain reaction (PCR) showed that the expressions of the 3 autophagy pathway genes were significantly upregulated in 1 μM and 2 μM cotinine treatment groups (*ATG5*: 1.83 and 2.27 folds; *BECN1*: 2.85 and 3.11 folds; *MAP1LC3B*: 1.81 and 2.61 folds, respectively), compared to the vehicle control group (*p* < 0.05; Fig. 6B). Moreover, 2 μM nicotine-cotinine treatment group showed an increased expression in *ATG5* and *BECN1* genes (1.84 and 1.98 folds, respectively; *p* < 0.05), but not *MAP1LC3B* gene. The results suggested that cotinine treatment could promote autophagy in RPE cells.

## Discussion

In the current study, our results showed that (1) cotinine reduces RPE cell proliferation; (2) nicotine and cotinine did not cause RPE cell apoptosis; (3) nicotine and cotinine did not affect RPE cell integrity; (4) cotinine retards RPE cell migration and downregulates EMT marker expression; (5) cotinine reduces pro-angiogenic factor and enhances anti-angiogenic factor expression; (6) nicotine and cotinine attenuate RPE phagocytotic activity; (7) cotinine increases autophagy pathway gene expression in RPE cells. Collectively, these data suggest that cotinine reduces RPE cell repair, wound healing ability and phagocytotic activity, which could be related to the pathological changes in AMD^2, 4, 5^. However, cotinine might not be responsible for releasing pro-angiogenic factors to stimulate the proliferation and invasion of choroidal capillaries in CNV.

There are over 4,000 chemicals in the cigarette smoke. We focused on nicotine because it is not only an important determinant factor for addiction^10^, but also the major component in cigarette replacements, such as electronic cigarette and nicotine patches, meaning that individuals may still be influenced under the process of quitting cigarette smoking. Under normal physiological conditions, nicotine absorbed into the bloodstream would be detoxified in the liver, where CYP2A6 catabolizes nicotine into cotinine^15^. The concentration of cotinine in the blood (1.02–1.73 μM) is higher than that of nicotine (0.08–0.15 μM) for daily cigarette smokers^17, 18^. Moreover, the half-life of cotinine (19 hours) is also longer than that of nicotine (2 hours)^16^. With potent biological activities, cotinine would affect the human system stronger than nicotine.

Previous studies have focused on the effects of nicotine on RPE cells. Neither low (10 nM) nor high concentration (10 mM) of nicotine treatment for 1–7 days affects RPE cell viability, proliferation or apoptosis^11–14^. Instead, exposure of 1 µM nicotine for 3 days changes RPE morphology^11^, whereas exposure of 100 µM nicotine for 7 days reduces the phagocytotic ability of the RPE^14^. Besides, nicotine would also reduce the expression of IL-8, PEDF, MMP2 and MMP9^11, 13^. In contrast, nicotine would upregulates VEGF in 10 nM concentration^12^, but decreases or shows no change in higher concentrations (1–100 µM)^11, 14^. Similarly, our study confirmed that 1 μM or 2 μM nicotine did not affect ARPE-19 cell viability, apoptosis and migration (Fig. 2 and Fig. 3A). Instead, 1 μM nicotine significantly attenuated RPE phagocytotic activity (Fig. 5). In addition, nicotine treatment did not disrupt RPE cell integrity (Fig. 2C) nor influence the angiogenic factor expression profile, except that VEGF expression is significantly reduced in the 2 μM nicotine treatment group (Fig. 4).

On the contrary, our results showed that, instead of nicotine, cotinine and nicotine-cotinine mixture induce the reduction in RPE cell proliferation and migration (Fig. 2A and Fig. 3A). The latter is associated with decreased protein expression of α-SMA and VIMENTIN, which is coherent to our previous observation in RPE cells under oxidative stress^22^. The reduction in cell proliferation by cotinine is not due to RPE cell apoptosis as there is no significant difference in sub-G1 population between the cotinine treatments and the vehicle control (Fig. 2B). Immunofluorescence analysis of ZO-1 (Fig. 2C) confirmed that RPE cell number and integrity are not affected by the cotinine treatment. We postulated that the reduction in cell proliferation by cotinine should be due to autophagy since increase in autophagic activity is associated with the suppression of ARPE-19 cell proliferation and the AMD pathology^4, 24, 25^. Upregulation in autophagy also links with the reduction of ARPE-19 cell viability by cigarette smoking extract^26^. In our study, the autophagy pathway genes, such as *ATG5*, *BECN1* and *MAP1LC3B*, are significantly upregulated in the cotinine treatment (Fig. 6B), indicating that the reduced RPE cell viability by cotinine could be associated with the upregulation in autophagy pathway. Moreover, the increase in autophagy can lead to decreased mitochondrial activity by mitophagy in RPE cells^27^, which would further reduce the cell viability signal. However, the mechanism of the cell viability reduction needs to be further verified.

In our attempts to delineate the angiogenic factor expression profile of cotinine-treated ARPE-19 cells, we found that cotinine treatment would decrease pro-angiogenic factor VEGF and IL-8 expression and increase anti-angiogenic factor TIMP-2 expression (Fig. 4), suggesting that cotinine favors anti-angiogenesis. In contrast, cotinine has been reported to enhance expression of VEGF in human and porcine endothelial cells as well as mouse hippocampus^28–30^. However, VEGF level is not altered by cotinine in human trophoblast cells^30^. Cotinine effects could be cell-type specific, consistent with the observations that nicotine could increase VEGF expression in endothelial cells and vascular smooth muscle cells^28, 30, 31^, but not in RPE cells^11, 13, 14^. Nevertheless, results in this study provide the first report on the correlation of cotinine with IL-8 and TIMP-2.

In summary, it is the first study to characterize the effect of cotinine, the major nicotine metabolite, on RPE cells *in vitro*. Cotinine reduces ARPE-19 cell repair, wound healing ability, phagocytotic activity as well as EMT marker, VEGF and IL-8 expression, but increases TIMP-2 and autophagy pathway gene expression, which could be related to the smoking-induced RPE pathology. Therefore, in our cell culture studies, cotinine exerted much more biological effects than nicotine. We speculate that cotinine plays a more prominent role than nicotine, in cigarette smoke. Results in this study also advocate further investigation on the effect of the metabolites of other toxic chemicals in the cigarette smoke on RPE cell functions.

## Materials and Methods

### Retinal pigment epithelial cell culture

Human RPE cell line (ARPE-19; CRL-2302) from American Type Culture Collection (Manassas, VA) has been established previously^32–34^. They were maintained and expanded in Dulbecco’s modified Eagle’s medium and F-12 nutrient mixture (DF-12 culture medium; Gibco BRL, Rockville, MD) supplemented with 10% heat-inactivated fetal bovine serum (Gibco BRL) and 1x penicillin/streptomycin (Gibco BRL) at 37 °C in 5% CO_2_. The cells used for all of the experiments in this study were within 5 passages. Each experiment was repeated at least 3 times.

### Nicotine and cotinine treatment

(−)-Nicotine (N3876; Sigma-Aldrich, St. Louis, MO) and (−)-cotinine (C5923; Sigma-Aldrich) were dissolved in dimethyl sulfoxide (DMSO). ARPE-19 cells or RPE explants were treated with 1 or 2 μM nicotine, cotinine or nicotine-cotinine mixture in DF-12 culture medium (final concentration of DMSO was 0.1%) for 5–7 days. The control group was the cells treated with 0.1% DMSO in DF-12 culture medium. The medium was changed daily in order to maintain a constant level of nicotine and cotinine.

### Cell proliferation analysis

ARPE-19 cell proliferation was assessed by MTT assay (Invitrogen, Carlsbad, CA) based on our previously established procedures^35, 36^. Briefly, 5,000 cells per well were seeded on a 24-well plate (Corning Life Sciences, Lowell, MA) and treated with nicotine and/or cotinine for 7 days. The analysis (3 wells per sample) was performed on Day 0, 1, 3, 5 and 7. Each sample was incubated with 0.05 mg/ml MTT reagent for 3 hours. After washing out excessive MTT reagent, the purple precipitates were dissolved in isopropanol and transferred to a 96-well plate (Corning Life Sciences) for intensity measurement. The absorbance at wavelength 570 nm with reference 650 nm was measured by a plate reader (Powerwave XS, Bio-Tek Instruments). The percentage of cell viability was determined as OD_570_ sample/OD_570_ control ×100%.

### Cell apoptosis analysis

ARPE-19 cell apoptosis was evaluated by propidium iodide (PI) staining with the flow cytometry analysis. Briefly, 2 × 10^5^ cells were seeded on a 60-mm dish (Corning Life Sciences) and treated with nicotine and/or cotinine for 7 days. Cells in the supernatant were collected every day when the medium was changed, and they were immediately fixed with 70% ethanol at 4 °C for at least 24 hours. Together with the trypsinized cells at Day 7, the fixed ARPE-19 cells were treated with 50 μl of 100 μg/ml RNase A (Pure link^TM^, Invitrogen) and 950 μl of 50 μg/ml PI (Sigma-Aldrich) in PBS for 20 mins at room temperature in dark. The cells were then passed through a 35 μm cell strainer (Falcon, Corning, NY). 1 × 10^5^ cells for each sample were analyzed by the flow cytometry machine (Cytomics FC500; Beckman Coulter, Indianapolis, IN). Single cells were identified using forward scatter (FS) and side scatter (SS). Cell clumps or doublets were excluded using the pulse area versus the peak height. The distributions of different cell cycle phases were identified using the cell counts (excitation: 488 nm, emission: 620 nm) versus the intensity of PI.

### Cell integrity analysis

ARPE-19 cell integrity was examined by the immunofluorescence analysis of tight junction protein (ZO-1) using our previously established protocol^37^. Briefly, 1 × 10^4^ cells per well were seeded on a glass coverslip in a 12-well plate (Corning Life Sciences). After 7-day nicotine and/or cotinine treatment, the cells were fixed in 4% paraformaldehyde (Sigma-Aldrich) for 15 min. After permeation and blocking, the treated ARPE-19 cells were labeled with primary mouse monoclonal antibodies against ZO-1 (BD Biosciences, San Jose, CA; Supplementary Table 1) for 18 hours at 4 °C, then with secondary antibody against mouse IgG conjugated with Alexa Fluor®488 (Santa Cruz Biotechnology, Dallas, TX; Dilution factor: 1:2000, working concentration: 0.2 ng/µl) for 1 hour at room temperature. The stained cells were mounted, and the fluorescence signals were visualized under a fluorescence microscope (Eclipse Ni-U; Nikon).

### Cell migration analysis

ARPE-19 cell migration was evaluated by the scratch wound assay. Briefly, 1 × 10^5^ cells per well were seeded on a 12-well plate. After 7-day nicotine and/or cotinine treatment, scratch wounds were created with 200-μl pipette tips on the pre-seeded confluence cells. The culture was washed after scratch wound induction and replaced by fresh medium. Photomicrographs were taken at time 0 (immediately following the scratch wound), 6, 12 and 24 hours. The wound gaps were measured by ImageJ (version 1.47; NIH, Bethesda, MD). The percentage migration was calculated by the average area reduction at 6, 12 or 24-hour as compared to time 0. Every well have 6 scratch wounds.

### Phagocytotic activity analysis

RPE explant culture was used to evaluate the phagocytotic activity of RPE cells *ex vivo* ^22^. Briefly, Sprague Dawley (SD) rats, aged 6–8 weeks, were enucleated and dissected. The cornea, iris and lens were first removed. The retina was then separated and collected for the photoreceptor outer segment (POS) processing. The remaining sclera explant, consisting of RPE, Bruch’s membrane, choroid and sclera, was cultured in DF-12 culture medium and treated with nicotine and/or cotinine for 5 days. All rats were treated according to the guidelines of the ARVO Statement for the Use of Animals in Ophthalmic and Vision Research. The experimental protocol was approved by the Animal Experimentation Ethics Committee of the Chinese University of Hong Kong.

FITC-labeled POS was prepared with the following procedures. Ten retinas from SD rats were dissected and digested in Trypsin-EDTA (Gibco BRL) at 37 °C for 15 min. 1 mg/ml Trypsin inhibitor (Invitrogen) was added and centrifuged for 5 min at 1,500 rpm. The cell pellet was vigorously re-suspended in 5 ml of homogenization buffer (34% sucrose, 65 mM NaCl, 2 mM MgCl_2_) and centrifuged for 4 min at 3,800 rpm. POS was separated from the cell body through vigorous resuspension, and the supernatant was diluted in 10 ml of 10 mM HEPES. The mixture was further centrifuged for 4 min at 3,800 rpm, and the resultant pellet, consisting of the POS fragments, was re-suspended in 1 ml of 10 mM HEPES. The protein concentration was determined by total protein assay (BioRad). 1 mg POS protein was agitated with 10 µl FITC-labelled latex beads (Sigma, 1 um in diameter) for 1 hour at room temperature. The opsonized beads were washed in 0.9% NaCl twice and re-suspended in 80 µl NaCl.

Phagocytotic activity was assessed after 5-day treatment of nicotine and/or cotinine. Briefly, the 1 ul of FITC-labeled latex beads with POS opsonization were added to each RPE explant and cultured for 6 hours. The explant was then washed twice with PBS before fixation in 4% paraformaldehyde (Sigma-Aldrich) for overnight at 4 °C. After permeation and blocking, the explant was stained with the primary mouse monoclonal antibody against RPE65 for 18 hr at 4 °C, then with secondary antibody anti-mouse IgG conjugated with Rhodamine RedX (Invitrogen). The stained explant was imaged using a confocal microscope (A1MP, Nikon). Sixteen fields were imaged for each explant, and 200 cells were counted in each field. Phagocytotic activity was quantified by the number of phagocytosed beads per cells.

### Angiogenic factor expression

Angiogenic factor profiling was performed using a multiplex ELISA array (Quansys Biosciences, Logan, UT), which can quantify the expression levels of 9 human angiogenic factors (ANG-2, FGF-2, HGF, PDGF, IL-8, TIMP-1, TIMP-2, TNFα and VEGF). Briefly, 2 × 10^5^ cells were seeded on a 60-mm dish and treated with nicotine and/or cotinine for 7 days. Supernatant was collected at Day 7, subjected to the multiplex ELISA array, and incubated at room temperature for 1 hour. After washing with the Wash Buffer for 3 times, Detection Mix was added to the plate and incubated at room temperature for 1 hour. After 3-time washing, horse radish peroxidase-conjudated Streptavidin was applied and incubated at room temperature for 15 minutes. After 6-time washing, the substrate was added, and the plate was imaged immediately using the ChemiDoc^TM^ XRS^+^ system (BioRad, Hercules, CA). The intensities of the spots were quantified and calculated by the Q-View software (Quansys Biosciences) according to the standard curves for each factor. Mean of the 6 repeats for each group was compared for the statistical significance.

### Gene and protein expression analysis

ARPE-19 cells (2 × 10^5^) were seeded on a 60-mm dish and treated with nicotine and/or cotinine for 7 days. To delineate the mechanism of the effects of nicotine and cotinine on RPE cells, the protein expression of EMT (α-SMA, SNAIL and VIMENTIN) and ER stress response markers (ATF6, GRP78 and PERK) was analyzed by immunoblotting with respective antibodies (Supplementary Table 1), whereas the expression of nicotinic acetylcholine receptor subunit and autophagy pathway genes (*ATG5*, *BECN1* and *MAP1LC3B*) was analyzed by semi-quantitative PCR with specific primers (Supplementary Table 2).

For the immunoblotting analysis, the treated cells were lysed by RIPA buffer (Sigma-Aldrich) supplemented with protease and phosphatase inhibitors (Roche). The total protein concentrations of the cell lysates were measured by Protein assay (BioRad). Equal amount of total protein (20 µg) for each denatured samples were resolved on 12.5% SDS-polyacrylamide gel and electro-transferred to nitrocellulose membranes for sequential probing with the primary antibodies and secondary antibodies conjugated with horseradish peroxidase (Santa Cruz Biotechnology; Dilution factor: 1:2000, working concentration: 0.2 ng/µl). The signals were detected by enhanced chemiluminescence (Amersham Pharmacia, Cleveland, OH) with the ChemiDoc^TM^ XRS^+^ system (BioRad). β-actin was used as housekeeping protein for normalization.

For the gene expression analysis, total RNA was extracted and purified with the TRIzol reagent according to the manufacturer’s protocol (Invitrogen, Carlsbad, CA). 1 µg of total RNA was reverse-transcribed by SuperScript® III reverse transcriptase (Invitrogen). The expression of nicotinic acetylcholine receptor subunit and autophagy pathway genes was evaluated by semi-quantitative PCR. Housekeeping gene (*GAPDH*) was used for normalization. The relative expression levels were compared to that of the vehicle control group.

### Statistical analysis

One-way analysis of variance (ANOVA) with post-hoc Tukey’s test (for multiple testing correction) was used to compare means among different treatment groups. All statistical analyses were performed by commercially available software (SPSS, version 16.0; SPSS Inc., Chicago, IL). Significance was defined as *p* < 0.05.

## Electronic supplementary material

Supplementary Information
