# Supplementary material for: Disruption of retinal pigment epithelial cell properties under the exposure of cotinine
Source: Sci Rep. 2017 Jun 9;7:3139. doi: 10.1038/s41598-017-03283-x (PMC5466671; doi:10.1038/s41598-017-03283-x)
Supplement: Supplementary file 1 — Supplementary Information [file 41598_2017_3283_MOESM1_ESM.pdf]

Disruption of retinal pigment epithelial cell properties under the exposure of cotinine

Xiao-Yu Zhang,[1,2,3,*] Tsz Kin Ng,[1,*] Mårten Erik Brelén,[1] Kwok Ping Chan,[1] Di Wu,[2,3] Jasmine Sum Yee Yung,[1] Di Cao,[1] Yumeng Wang,[1] Shaodan Zhang,[2,3] Sun On Chan,[4] Chi Pui Pang.[1]

[1] Department of Ophthalmology and Visual Sciences, and [4] School of Biomedical Sciences, The Chinese University of Hong Kong, Hong Kong

[2] Department of Ophthalmology, The Fourth People's Hospital of Shenyang, and [3] Shenyang Key Laboratory of Ophthalmology, Shenyang, China

* Co-first author

Correspondence:

Tsz Kin Ng, PhD

Department of Ophthalmology and Visual Sciences, The Chinese University of Hong Kong

4/F, Hong Kong Eye Hospital, 147K Argyle Street, Kowloon, Hong Kong

Phone: +852-39435809; FAX: +852-27159490; E-mail: micntk@hotmail.com

Supplementary Table 1: Antibodies for protein expression analysis.

Protein	Company	Catalog number	Source	Dilution factor (Working concentration)
ZO-1	BD Biosciences	610966	mouse	1:500 (0.5 ng/µl)
α-SMA	DAKO	M0851	mouse	1:500 (0.5 ng/µl)
SNAIL	Abcam	ab180714	rabbit	1:500 (2 ng/µl)
VIMENTIN	DAKO	M7020	mouse	1:1000 (0.25 ng/µl)
GRP78	BD Biosciences	610979	mouse	1:500 (0.5 ng/µl)
ATF6	Imgenex	IMG-273	mouse	1:500 (2 ng/µl)
PERK	Santa Cruz Biotechnology	sc-13073	rabbit	1:500 (0.4 ng/µl)
RPE65	Millipore	MAB5428	mouse	1:500 (2 ng/µl)
β-ACTIN	Sigma-Aldrich	A3854	mouse	1:2000 (1 ng/µl)

Supplementary Table 2: Primers for gene expression analysis.

Gene		Primer Sequence (5' > 3')	Tm (℃)	Accession number
CHRNA1	*F:*	GTGCGTCTGAAACAGCAATGG	60	NM_000079.3
	R:	GTACTGCAGGAGCACTTTGGT		
CHRNA2	*F:*	TCGACGGGGTGTCTCCTAAA	60	NM_000742.3
	R:	CACCGAACCTGTGGTTACAGA		
CHRNA3	*F:*	CCGTCTATTTGAGCGGCTGT	60	NM_000743.4
	R:	TCCAGATTTGCTTGAGCCACA		
CHRNA4	*F:*	GTCCTCTACAACAATGCTGACG	60	NM_000744.6
	R:	GGTCCAGGAGCCGAATTTCA		
CHRNA5	*F:*	AAACGTCTGGTTGAAACAGGAATG	60	NM_000745.3
	R:	TGACAGTGCCATTGTACCTGA		
CHRNA6	*F:*	GCCAACGTGGATGAAGTAAACC	60	NM_004198.3
	R:	CAGGAACGCGAAGAGTCTCA		
CHRNA7	*F:*	CTGCACGTGTCCCTGCAA	60	NM_000746.5
	R:	CGTCCATGATCTGCAGGAGG		
CHRNA9	*F:*	GAAAATGTGCCCCTGATAGGTAAA	60	NM_017581.3
	R:	AGACAAACAAGACCCTGGACA		
CHRNA10	*F:*	TCATCGACATGGATGAACGGAAC	60	NM_020402.3
	R:	TGCGCGTCGGCTTTGTTA		
CHRNB1	*F:*	ACTTAGACCTGGAGTGGACTGA	60	NM_000747.2
	R:	GAGGACACCACGACGCTAAT		
CHRNB2	*F:*	ACAACAAGCTTATCCGCCCA	60	NM_000748.2
	R:	ATCTTCCCACTCCTGGGTCA		
CHRNB3	*F:*	CCTCTTGGGTTCCACTTCGG	60	NM_000749.3
	R:	TTGAAACCTGTGGTGGCTGA		
CHRNB4	*F:*	TTTCTTCCTGGTCGCCCTTTG	60	NM_000750.3
	R:	CTCTCGCTCATTCACGCTGATAA		
CHRND	*F:*	CCGGCACCTGTTTCAAGAGA	60	NM_000751.2
	R:	TCCAGCCGTGCTCTATCCA		
CHRNE	*F:*	GCGGAGGAGCTGATACTGAAA	60	NM_000080.3
	R:	CAGTCGGACACTTCCTCGC		
CHRNG	*F:*	TGGATAGAGATGCAGTGGTGC	60	NM_005199.4
	R:	TAGATACAGCCGTCAGGGGA		
ATG5	*F:*	GCCATAGCTTGGAGTAGGTTTG	60	NM_004849.3
	R:	CGTCCAAACCACACATCTCG		

BECN1	*F:*	CCGAGGTGAAGAGCATCGG	60	NM_003766.4
	R:	TGTGGTAAGTAATGGAGCTGTGA		
MAP1LC3B	*F:*	TGCGGGCTGAGGAGATACA	60	NM_022818.4
	R:	TCTACTCTTTGTTCGAAGGTGCG		
GAPDH	*F:*	TGTTGCCATCAATGACCCCTT	60	NM_002046.3
	R:	CTCCACGACGTACTCAGCG		
